# Supplementary material for: Long-range focusing of magnetic bound states in superconducting lanthanum
Source: Nat Commun. 2020 Sep 11;11:4573. doi: 10.1038/s41467-020-18406-8 (PMC7486372; doi:10.1038/s41467-020-18406-8)
Supplement: Supplementary file 1 — Supplementary Information [file 41467_2020_18406_MOESM1_ESM.pdf]

Long-range focusing of magnetic bound states in superconducting lanthanum

Kim *et al.*

Supplementary Note 1. Thickness-dependent surface electronic structure of La(0001).

The La islands grown on the Re(0001) surface with different thicknesses are shown in Supplementary Figures 1a and 1b. The Stranski-Krastanov growth leads to the formation of the La thin films directly above the monoatomic La wetting layer on the clean Re(0001) surface. The top surface of the La thin films is atomically flat and terminated by an (0001)-oriented surface plane following the crystal structure of Re.

To explore the surface electronic structure, we took tunneling spectra on the La(0001) surface of the island with different thicknesses and on the La wetting layer (WL), as shown in Supplementary Figure 1c. We observed sharp resonances on the La(0001) surface for both thin films at V=110 mV, which is the signature of the so-called Tamm-type surface states originating from the d_{z^2}-like atomic orbital of lanthanum[1]. Note that Tamm-type surface states show a strong resonance peak at the edge of the surface band, instead of displaying a step-like LDOS characteristic for Shockley surface states, which originate from a delocalized *sp*-band. On the other hand, the La wetting layer shows a featureless LDOS within this energy window. Interestingly, the thicker La film shows a sharper and higher resonance peak, indicating that the coupling with the Re substrate becomes weak for the thick La film. The resonance peak of the Tamm-type surface states at E_S=0.11 eV is consistently reproduced by the LDOS determined from ab initio calculations in the vacuum 3 Å above the surface of bulk La as shown in the inset of Supplementary Fig. 1c. Supplementary Figure 1d shows the tunneling spectra around the Fermi energy obtained on the top surfaces of the La films of two different thicknesses and on the La wetting layer. The thicker film (55.0 nm) shows a clear BCS-type superconducting spectral shape, with the superconducting gap (Δ_{La}) of 0.95 meV, while the thinner film (12.5 nm) shows a non-BCS-type superconducting spectral shape with peaks at V=±0.95 mV and shoulders at V=±0.30 mV. The La wetting layer displays a BCS-type superconducting spectrum with a superconducting gap of 0.30 meV, which is similar to the gap size of bare Re(0001). This implies that the shoulders in the spectra for the thinner La layer are a contribution of the Re(0001) substrate through the superconducting proximity effect, and the peaks at ±0.95 mV are the edges of the intrinsic superconducting gap of La. Since the coherence length of bulk La is about 36.3 nm, and the thickness of the thinner island is below this value, the proximity effect suppresses the superconducting nature of the La island. For the 55.0 nm thick film we do not observe any signature of multi-gap superconductivity in our spectroscopic measurements within the energy resolution at the given experimental temperature. These

results support the treatment of the thicker La film grown on Re(0001) as bulk La with an isotropic superconducting gap, as was done in the main text.

Supplementary Note 2. Identification of the subsurface magnetic impurities.

As described in the main text, the magnetic impurities were co-deposited during the growth of the La films on the Re(0001) substrate. A constant-current image of the surface containing several impurities is shown in Supplementary Figure 2a. To investigate the adsorption sites of the magnetic impurities, we applied the STM-induced atom-manipulation technique[2]. At first we were not able to laterally manipulate the impurities on the surface with typical atom-manipulation parameters (bias voltages ~1 mV, tunneling set current > 500 nA). However, by releasing a single La atom from the STM tip apex and placing it between the tip and the surface during the imaging, we managed to obtain an STM manipulation image over the magnetic impurities, see Supplementary Figure 2b. Remarkably, although the magnetic impurities can be identified in the constant-current and the *dI/dV* images (Supplementary Figure 2c) by the long-range extension of the YSR bound states, we observe a defect-free continuous atomic lattice of the surface La atoms in the atom manipulation image at those sites; this is indicated by the dotted circles highlighting the same area in Supplementary Figures 2a-2c. This observation clearly indicates that the magnetic impurities are not located within the top surface plane, but they are embedded below the surface of the La(0001) films.

Supplementary Note 3. Various subsurface YSR impurities in the La(0001) film

As described in the main text, we primarily observe four different types of YSR impurities (YSR1-YSR4) in our La(0001) film. Although we have mainly focused on the YSR1-type impurities showing a strong peak intensity at $E=E_{\mathrm{YSR}}$ in the spectrum and a remarkably long-range extension of the YSR bound states in real space, we present the typical spectra for all four types of YSR impurities in Supplementary Figure 3. From the analysis of the spectral shapes for all YSR impurities, we found that the extracted YSR binding energies are 0.52 meV, 0.11 meV, 0.40 meV, and 0.97 ($\sim\Delta_{\mathrm{La}}$) meV for YSR1, YSR2, YSR3, and YSR4, respectively. Interestingly, YSR1 and YSR2 show pronounced peaks at $E=\pm E_{\mathrm{YSR}}$, while YSR3 and YSR4 show weak spectral weights, leading to an almost similar spectrum as obtained for the defect-free region of the La(0001) surface. Indeed, the spatial modulations of YSR bound states for

YSR3 and YSR4 are quite weak in the *dI/dV* map as shown in Fig. 1a compared to the LDOS modulations for YSR1 and YSR2. This may indicate that the YSR3 and YSR4 impurities are probably located deeper in the La film than the YSR1 and YSR2 impurities. Therefore, the various shapes of the modulations (YSR1, YSR2, YSR3, and YSR4 in Figs. 1a,b) and their spectral variations presumably originate from the various depths of the magnetic impurities [see Supplementary Note 2] or different magnetic elements being present as natural impurities in the La film as discussed in the main text.

Supplementary Note 4. Quasiparticle scattering of surface electrons.

The standing-wave patterns produced by the electron scattering around the surface defects provide information about the dispersion of the surface band structure via energy-dependent quasiparticle interference (QPI) imaging followed by Fourier transformation of the real-space data. Supplementary Figures 4a and 4b show the STM constant-current image and simultaneously measured *dI/dV* image around the Fermi energy (*E*=+3 meV), visualizing the QPI on the La(0001) surface. The Fourier transformed (FT) QPI images (Supplementary Figure 4c) clearly reveal the scattering vectors forming nearly hexagonal shapes due to the anisotropic electron scattering on the La(0001) surface. The length of the scattering vectors is energy dependent; at decreasing bias voltage, the size of the hexagon is increased, which means that the magnitude of the scattering vectors is increased. The hexagonal shape of the FT QPI patterns and their dispersion are in strong contrast to the case of the *sp*-band induced Shockley surface states of noble metals such as Au, Ag and Cu, which display circular patterns and an increased magnitude of the scattering vectors at higher energy[3]. We quantitatively analyzed the FT QPI images to extract the scattering vectors for different directions and at different bias voltages, as shown in the right panel of Fig. 2a. Interestingly, although the surface band of La(0001) leads to quasi-2D electronic properties manifested in the extension of the YSR states, deviations from a 2D electron gas as resulting from isotropic surface bands with parabolic band dispersion occur. This difference mainly originates from the localized d-orbital character of the surface states of La(0001).

Supplementary Note 5. Spatial extent of the YSR bound states

As it has been calculated in refs. (4, 5), for an isotropic Fermi surface the spatial profile of the YSR wave function follows the asymptotic form

$$\psi_{\pm}(r) \propto (k_{\mathrm{F}} r)^{\frac{1-d}{2}} \sin(2\pi k_{\mathrm{F}} r + \varphi_{\pm})\, e^{-\frac{\sqrt{\Delta^2 - E_{\mathrm{YSR}}^2}}{\hbar v_{\mathrm{F}}} r}, \tag{1}$$

where d=2 or 3 is the dimension of the system, k_{F} is the Fermi wave vector, v_{F} is the Fermi velocity, and $\varphi_{\pm}$ are phase factors of the YSR states determined by the energy E_{YSR} and the dimensionality. The characteristic length scale of the exponential cut-off equals the coherence length $\xi = \frac{\hbar v_{\mathrm{F}}}{\Delta}$ for $E_{\mathrm{YSR}} = 0$, and is increased by a factor of $\Delta/\sqrt{\Delta^2 - E_{\mathrm{YSR}}^2}$ as the YSR energy approaches the coherence peak. Since the coherence length is on the order of tens of nanometers (36.3 nm in bulk La), the decay of the YSR state with distance observed in the STM measurements is dominated by the power-law prefactor, determined by the dimension of the system and the shape of the Fermi surface.

To elucidate the significant role of the shape of the Fermi surface in the spatial extent, we varied the normal-state dispersion relation $\xi_{\mathbf{k}}$ in Eq. (2) of the main text to interpolate between the circular and hexagonal limits, as illustrated in Supplementary Figure 7. The Fermi wave vector k_{F}=0.52 nm^{-1} and the Fermi velocity $\hbar v_{\mathrm{F}}$=1.18 eV·nm were kept fixed at their values determined from the *ab initio* calculations along the ΓM or **Q2** direction in the Brillouin zone. Six points were selected along the ΓK or **Q1** directions in reciprocal space, at a distance of $k_\alpha = \alpha k_{\mathrm{F}} 2/\sqrt{3}$ from the center, where α is the deformation parameter. Isoenergy surfaces were constructed by drawing circle segments centered on these points between angles –π/6 and π/6 measured from the ΓK direction, then connecting these segments by straight lines, which were perpendicular to the ΓM direction by construction. These surfaces are perfectly hexagonal for $k < k_\alpha$ and perfectly circular in the limit $k/k_\alpha \to \infty$, meaning that changing α between 0 and 1 interpolates the shape of the Fermi surface between these two limits. The value of $\frac{JS}{2}$ =0.46 meV was kept constant during the simulations. It was found that the deviation of E_{YSR} from the experimental value was kept below 10% while moving from the circular to the hexagonal Fermi surface (see Supplementary Movie 1), so it is expected that this does not influence the spatial decay considerably.

Supplementary Figures 5a to c show the calculated LDOS maps of the hole- and the electron-like YSR bound states ($|\psi_{\pm}(r)|^2$) for different shapes of the Fermi surface. In addition to the

focusing effect along the directions perpendicular to the flat parts of the Fermi surface (**Q2**), the plots demonstrate the same spatial extent at positive and negative energies, with a phase shift of $2(\varphi_+ - \varphi_-)$ between the oscillatory modulations.

In order to suppress the oscillatory factor in Eq. (1), the envelope functions of the LDOS were extracted from the simulations. As shown in Supplementary Figure 5d, these functions follow a power-law decay r^b for distances shorter than the coherence length. For the circular Fermi surface, the profile shows a 1/*r* decay for both the **Q1** and the **Q2** directions, characteristic for 2D systems as follows from the analytic formula Eq. (1). The focusing effect is captured by the two different exponents observed for the hexagonal Fermi surface: b<-1 for the **Q1** direction and b>-1 for the **Q2** direction, the latter approaching the zero value characteristic of one-dimensional systems. The exponent b allows to quantify the role of the deformation factor α for the spatial extent of the YSR states, as shown in Supplementary Figure 5e. As the Fermi surface becomes more anisotropic, b is gradually increased (decreased) along the **Q2** (**Q1**) direction. The anisotropic decay is also apparent for the envelope curves of the experimentally obtained YSR intensities displayed in Supplementary Figure 5f, with exponents deviating from b=-1 along both the **Q1** and the **Q2** directions. Note that the discrepancy in the numerical values of the exponents between the model and the experimental results is due to the fact that bulk contributions also have a minor influence on the YSR states formed at the subsurface magnetic impurities, which was neglected in the simulations.

Supplementary Note 6. Calculation of the YSR states.

The calculation of the YSR states based on the atomistic Hamiltonian given by Eq. (2) in the main text followed the procedure outlined in refs. (4, 5). The Bogoliubov operators b_q are introduced as

$$c_{\mathbf{k}\sigma} = \sum_q \left(u_{\mathbf{k}\sigma q} b_q + v^*_{\mathbf{k}\sigma q} b_q^\dagger\right), \qquad (2)$$

and they diagonalize the Hamiltonian,

$$\left[b_q, H\right] = \varepsilon_q b_q. \qquad (3)$$

The eigenvalues ε_q and the eigenvectors $\psi_q(\mathbf{k}) = \left(u_{\mathbf{k}\uparrow q}, v_{-\mathbf{k}\downarrow q}\right)$ satisfy

$$\varepsilon_q \psi_q(\mathbf{k}) = (\xi_{\mathbf{k}}\tau_z + \Delta\tau_x)\psi_q(\mathbf{k}) + \frac{1}{N}\left(-\frac{JS}{2} + K\tau_z\right)\sum_{\mathbf{k}'} \psi_q(\mathbf{k}), \tag{4}$$

or

$$\psi_q(\mathbf{r}) = \sum_{\mathbf{k}} e^{i\mathbf{kr}}\,\psi_q(\mathbf{k}) = \frac{1}{N}\sum_{\mathbf{k}} e^{i\mathbf{kr}} \frac{\varepsilon_q + \xi_{\mathbf{k}}\tau_z + \Delta\tau_x}{\varepsilon_q^2 - \xi_{\mathbf{k}}^2 - \Delta^2}\left(-\frac{JS}{2} + K\tau_z\right)\psi_q(\mathbf{r} = \mathbf{0}) \tag{5}$$

in real space. Equation (5) has a single bound state solution for which $|\varepsilon_q| < \Delta$. The eigenvalue may be calculated via fix-point iteration by setting $\mathbf{r} = \mathbf{0}$ on the left-hand side, and ε_q in turn enables determining the spatial profile of the wave function. The density of states is given by the particle part, LDOS= $|u_{\mathbf{k}\uparrow q}|^2$. Due to the particle-hole symmetry of the Hamiltonian, the hole part $v_{-\mathbf{k}\downarrow q}$ at ε_q corresponds to the particle part of the YSR state observed at the opposite bias voltage, having the eigenvalue $-\varepsilon_q$.

Supplementary Figure 1.

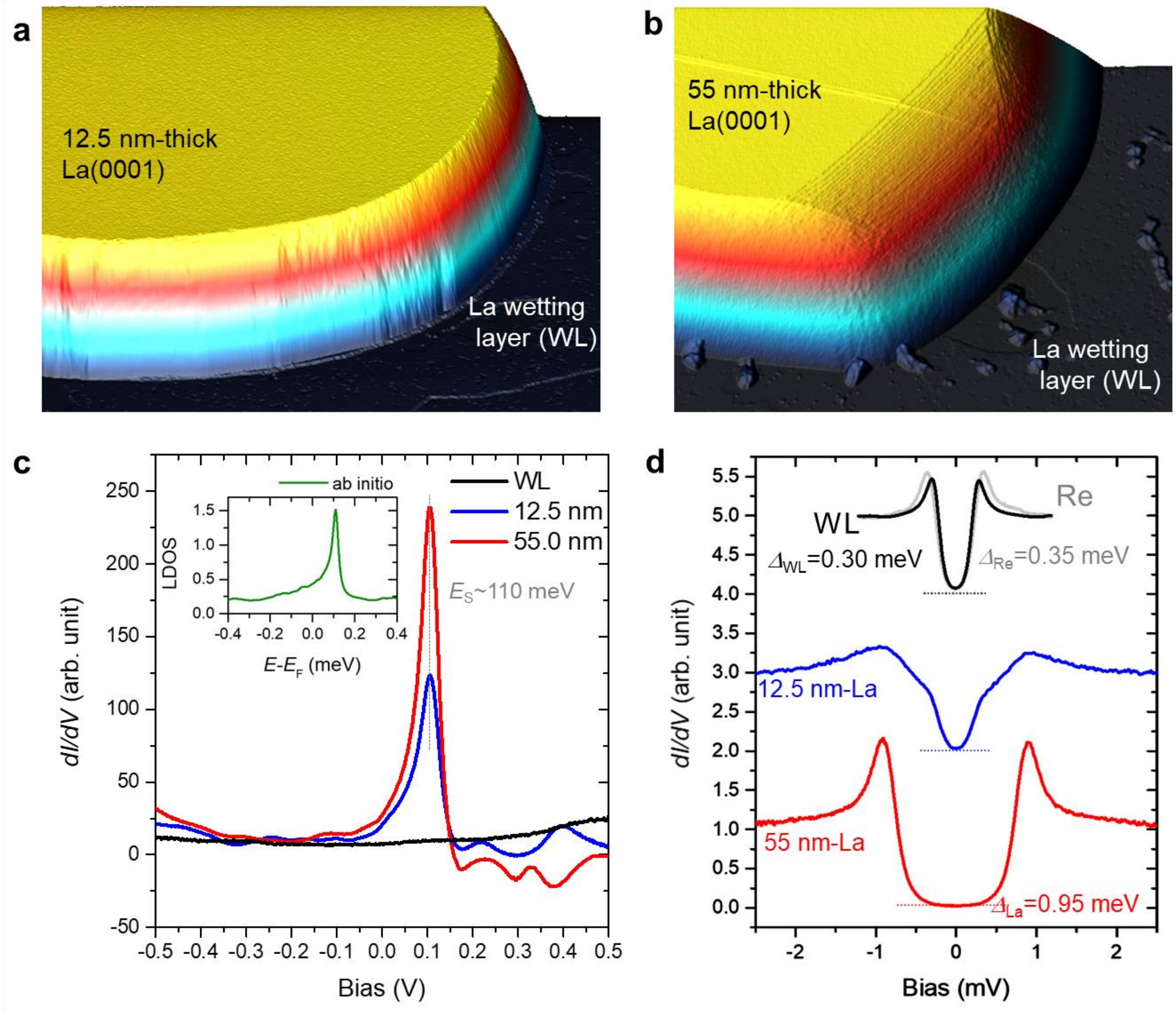

Supplementary Figure 1. Thickness-dependent electronic structure of La on Re(0001) at T=0.4 K.

a-b, Constant-current STM images of two La islands on the La wetting layer on Re(0001) with an island thickness of (a) 12.5 nm and (b) 55.0 nm (I=0.1 nA, V=100 mV). The surface of both La islands is terminated by a flat (0001)-plane. **c**, Typical tunneling spectra obtained above the La wetting layer (WL, black), the 12.5-nm-thick La island (blue) and the 55.0-nm-thick La island (red) as shown in **a** and **b**. (inset) Calculated LDOS in the vacuum 3 Å above the surface. Stabilized tunneling conditions: I_T=1.0 nA, V= 0.5 V. The grey dotted line indicates the peak position of the Tamm-type surface state at V=110 mV. **d**, Tunneling spectra obtained in the lower energy range for the La wetting layer and the two La islands. Stabilized tunneling conditions: I_T=1.0 nA, V=1.2 mV for the La wetting layer and I_T=1 nA, V= 2.5 mV for the La islands. Note that a PtIr tip was used for STM imaging and tunneling spectroscopic measurements.

Supplementary Figure 2.

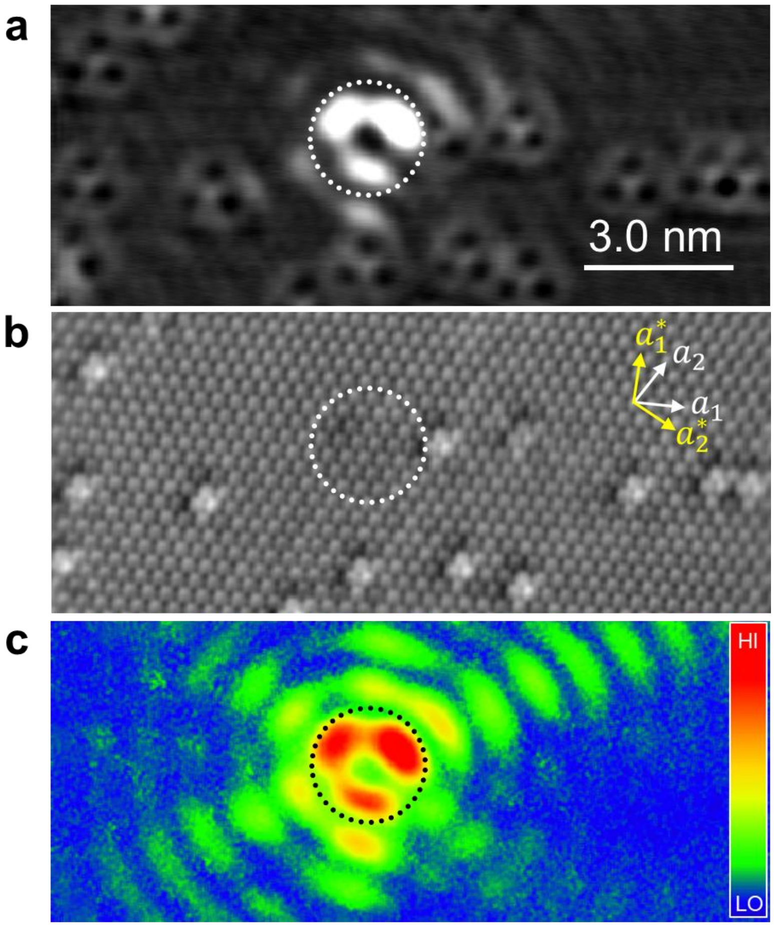

Supplementary Figure 2. Determination of the position of the magnetic YSR1 impurity.

a, Constant-current STM image of the isolated magnetic impurity at I_T=5.0 nA and V_S=+1.1 mV. (b) Atom-manipulation image of the same area as in **a**, obtained by dragging a single La atom over the surface by the STM tip at I=350 nA and V=3.0 mV. **c**, *dI/dV* map obtained simultaneously with **a**, showing the spatially-extended LDOS variation of the YSR1 bound state of the subsurface magnetic impurity. Dotted circles indicate the location of the magnetic impurity. A La-coated PtIr tip was used for STM imaging.

Supplementary Figure 3.

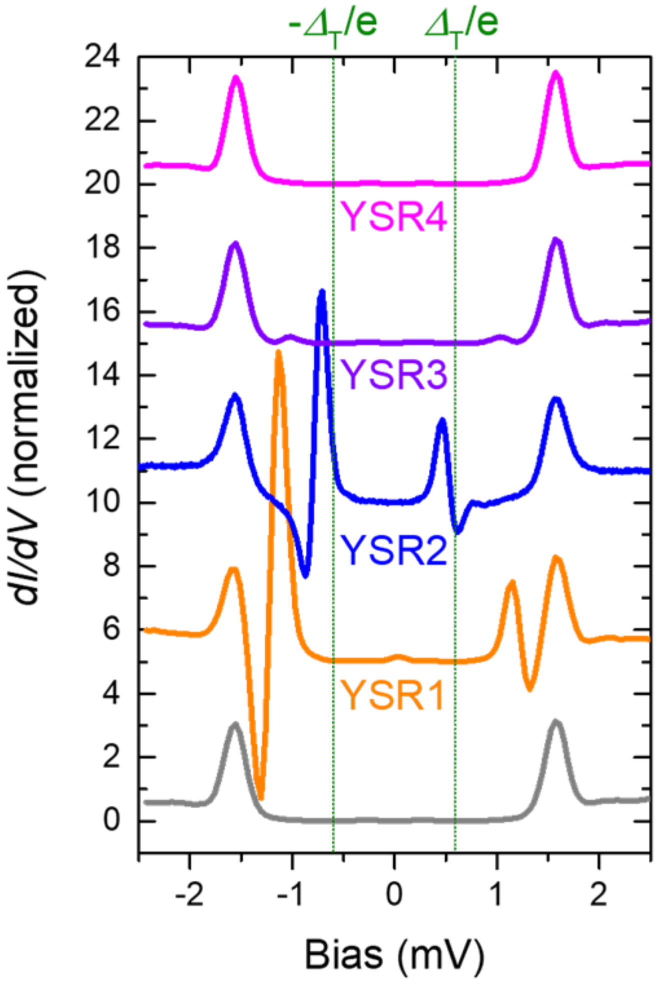

Supplementary Figure 3. Tunneling spectra for the YSR1-YSR4 impurities on La(0001).

Tunneling spectra obtained above the YSR1, YSR2, YSR3 and YSR4 impurities introduced in Fig. 1 of the main text, at T=1.65 K. The curves are vertically shifted for clarity. All spectra have been measured with a La-coated superconducting tip whose superconducting gap is 0.6 meV as marked by green dotted lines. For reference, a typical spectrum (gray curve) obtained at a far distance away from all YSR impurities on the pure La(0001) film is presented at the bottom. The stabilized tunneling conditions are I_T= 1.0 nA and V_S=3.0 mV.

Supplementary Figure 4.

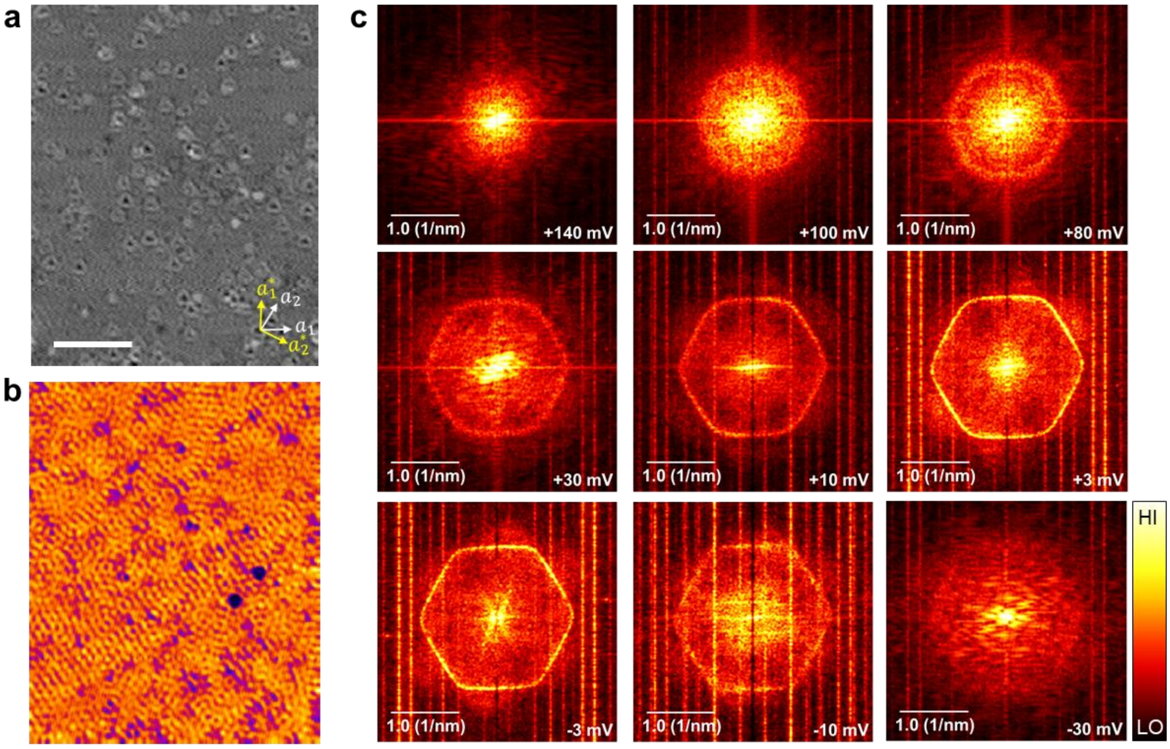

Supplementary Figure 4. Bias-dependent quasiparticle scattering of the La(0001) surface electrons.

a, STM constant-current images obtained on the La(0001) surface. **b**, Simultaneously obtained *dI/dV* maps at +3.0 mV **c**, Fourier transforms of the *dI/dV* maps obtained for various applied bias voltages, indicated in the lower right corner of each sub-image. The colour scales of all images were adjusted separately for better visibility. A La-coated PtIr tip was used for STM imaging with a stabilizing current of I_T=1.0 nA at T=1.65 K.

Supplementary Figure 5.

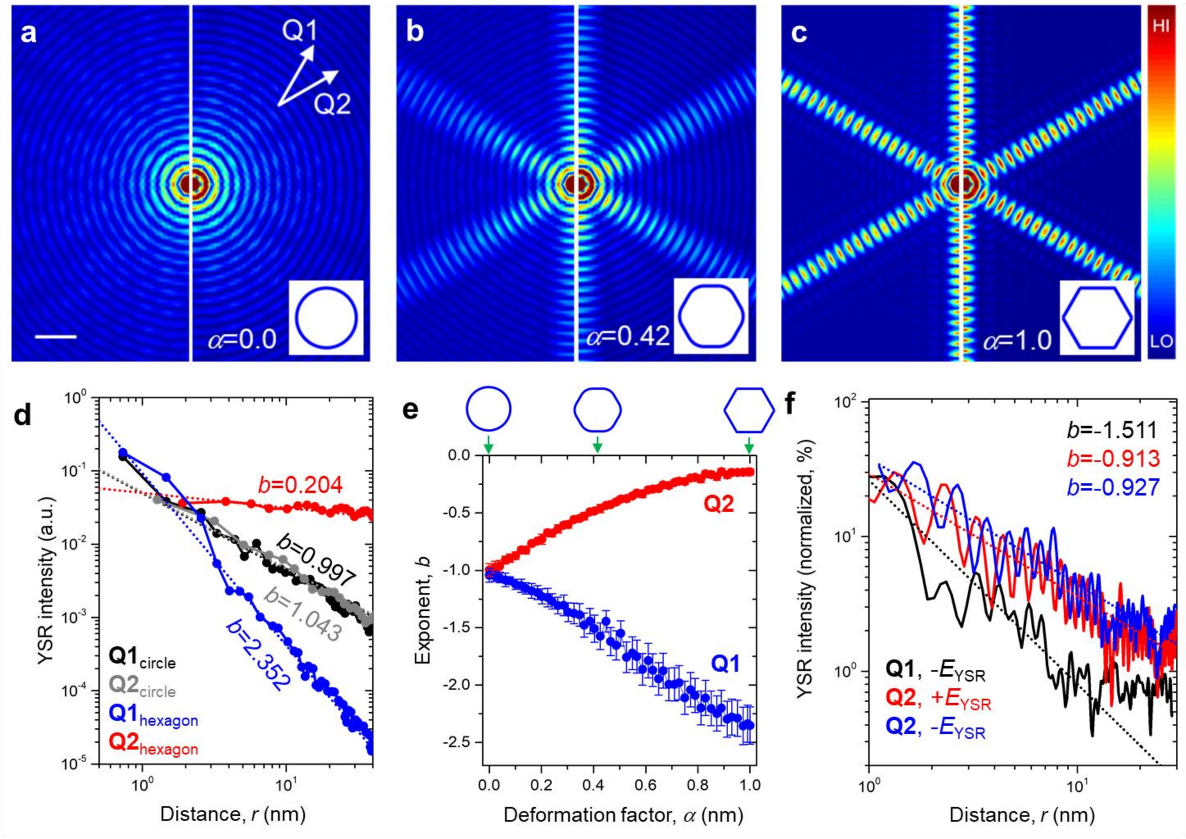

Supplementary Figure 5. Calculated LDOS of the YSR bound states depending on the shape of the Fermi surface.

Numerically calculated LDOS around a single magnetic impurity for the (left) hole- and the (right) electron-like YSR bound states, considering (**a**) circular and (**c**) hexagonal Fermi surfaces, as well as (**b**) an intermediate one constructed based on the first-principles calculations for the surface band on La(0001). The corresponding shape of the Fermi surface is depicted in the inset for each calculated image. Scale bar: 10 atomic sites=3.77 nm. **d**, Log-log plots of the LDOS envelope profiles of the hole-like YSR bound states as a function of distance from the impurity along the **Q1** and **Q2** directions, for the circular and the hexagonal Fermi surface. Dotted lines are fits to the power function ar^b. **e**, Dependence of the exponent b of the fitting function on the deformation factor α. The Fermi surfaces used for calculating panels **a**-**c** are indicated at the top of the figure. The error bars denote the standard deviation of the curve fitting for the parameter b. **f**, Experimentally obtained LDOS profiles of the YSR states along the **Q1** and the **Q2** directions. Dotted lines are fits to the power function ar^b.

Supplementary Figure 6.

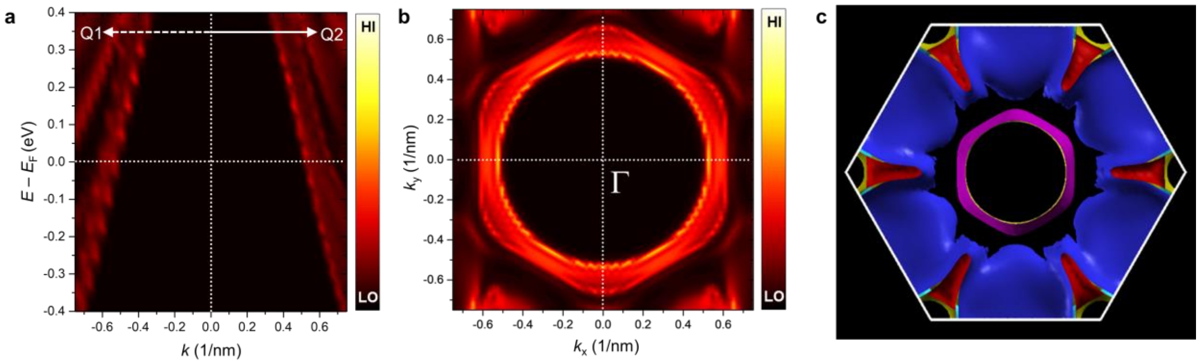

Supplementary Figure 6. Bloch spectral function in bulk La and Fermi surface.

Contour plot of the Bloch spectral function in the 12th atomic layer from the (0001) surface of La, along (**a**) the **Q1-Q2** directions (KΓM line) and (**b**) in the Brillouin zone at the Fermi energy. Compared to the data in the top surface layer shown in Figs. 2b and d, the flat band located at around 100 meV disappeared, and the inner hexagonal contour at the Fermi level is not visible. Therefore, these features may be assigned to a surface band. In the layer-resolved calculations, the spectral function is essentially integrated along the k_z direction, yielding broad features instead of sharp peaks for the bulk bands. For comparison, the Fermi surface of bulk La is shown in panel **c**[6], calculated using the SKKR method. This was obtained by calculating the Bloch spectral function in the three-dimensional Brillouin zone, and by finding the peaks as a function of energy at each **k** point to construct the dispersion relation. The same parameters were used for the self-consistent bulk calculations as for the layered calculations reported in the Methods, with an additional 4 k_z points along the [0001] direction.

Supplementary Figure 7.

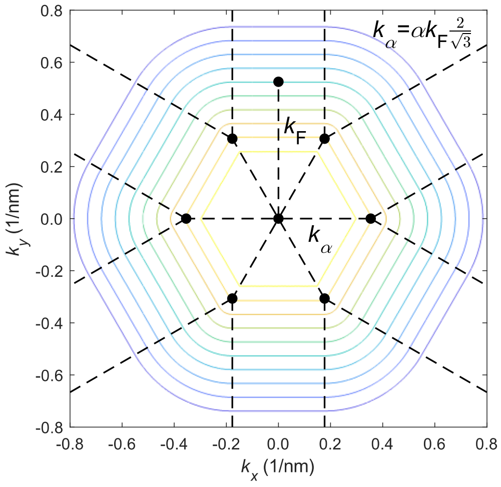

Supplementary Figure 7. Interpolation between circular and hexagonal Fermi surfaces in the atomistic model.

Contour plot of the normal-state dispersion relation $\xi_{\mathbf{k}}$ used for the model calculations, illustrating the construction of the model dispersion described in Supplementary Note 4 with the deformation parameter α. Dashed lines separate flat and circular parts of the isoenergy contours.

Supplementary Table 1.

ξ_0 (eV)	t_1 (eV)	t_2 (eV)	t_3 (eV)	t_4 (eV)	t_5 (eV)
-12.91	4.36	-2.60	-0.16	0.46	0.04

Supplementary Table 1. Tight-binding parameters for the surface band of La(0001), used for the calculation of the YSR states.

The dispersion relation obtained from the Bloch spectral function was fitted to a single-band tight-binding model on the triangular lattice containing hopping parameters up to the fifth neighbours,

$$\begin{aligned}\xi_{\mathbf{k}} = \xi_0 &+ 2t_1\left[2\cos\left(\frac{1}{2}k_x 2\pi a_0\right)\cos\left(\frac{\sqrt{3}}{2}k_y 2\pi a_0\right) + \cos(k_x 2\pi a_0)\right] \\ &+ 2t_2\left[2\cos\left(\frac{3}{2}k_x 2\pi a_0\right)\cos\left(\frac{\sqrt{3}}{2}k_y 2\pi a_0\right) + \cos\left(\sqrt{3}k_y 2\pi a_0\right)\right] \\ &+ 2t_3\left[2\cos(k_x 2\pi a_0)\cos\left(\sqrt{3}k_y 2\pi a_0\right) + \cos(2k_x 2\pi a_0)\right] \\ &+ 4t_4\left[\cos\left(\frac{1}{2}k_x 2\pi a_0\right)\cos\left(\frac{3\sqrt{3}}{2}k_y 2\pi a_0\right)\right. \\ &+ \cos\left(\frac{5}{2}k_x 2\pi a_0\right)\cos\left(\frac{\sqrt{3}}{2}k_y 2\pi a_0\right) \\ &\left.+ \cos(2k_x 2\pi a_0)\cos\left(\sqrt{3}k_y 2\pi a_0\right)\right] \\ &+ 2t_5\left[2\cos\left(\frac{3}{2}k_x 2\pi a_0\right)\cos\left(\frac{3\sqrt{3}}{2}k_y 2\pi a_0\right) + \cos(3k_x 2\pi a_0)\right]. \end{aligned} \quad (6)$$

Here k_x is along the $[1\bar{1}00]$ next-nearest-neighbour direction in the reciprocal lattice, while k_y is along the $[11\bar{2}0]$ nearest-neighbour direction. The numerical values of the parameters are listed in the table. Since the surface band merges with the bulk bands, the dispersion relation was only extracted in the energy region of E_F±27.2 meV around the Fermi level. Outside this regime, the tight-binding model is not representative of the band structure of La(0001), but it is appropriate for calculating superconducting properties since the energy window was significantly larger than the gap.

References

1. Wegner, D. *et al.* Surface electronic structures of La(0001) and Lu(0001). *Phys. Rev. B* **73**, 115403 (2006).
2. Stroscio, J. A. Controlling the Dynamics of a Single Atom in Lateral Atom Manipulation. *Science* **306**, 242–247 (2004).
3. Sessi, P. *et al.* Direct observation of many-body charge density oscillations in a two-dimensional electron gas. *Nat. Commun.* **6**, 8691 (2015).
4. Rusinov, A. I. Superconductivity Near a Paramagnetic Impurity. *JETP Lett.* **9**, 85–87 (1969).
5. Ménard, G. C. *et al.* Coherent long-range magnetic bound states in a superconductor. *Nat. Phys.* **11**, 1013–1016 (2015).
6. Kokalj, A. XCrySDen—a new program for displaying crystalline structures and electron densities. *J. Mol. Graph. Model.* **17**, 176–179 (1999).
