# Supplementary material for: Long-range focusing of magnetic bound states in superconducting lanthanum
Source: Nat Commun. 2020 Sep 11;11:4573. doi: 10.1038/s41467-020-18406-8 (PMC7486372; doi:10.1038/s41467-020-18406-8)
Supplement: Supplementary file 3 — Description of Additional Supplementary Files [file 41467_2020_18406_MOESM3_ESM.pdf]

Description of Additional Supplementary Files

Supplementary Movie 1:
"Calculated LDOS maps of the hole-like YSR bound state around a magnetic impurity as the Fermi surface is gradually modified from a circle to a hexagon. (top) Simulated Fermi surfaces considering the deformation parameter α [see Supplementary Note 5]. (bottom) Calculated LDOS maps of the YSR bound state considering the corresponding Fermi surface."
